# Using the Welsh Index of Multiple Deprivation in research: estimating the effect of excluding domains on a routine health data study

**DOI:** 10.1186/s12889-025-22369-0

**Published:** 2025-03-28

**Authors:** Shamsudeen Mohammed, Grace A. Bailey, Ian W. Farr, Carys Jones, Anna Rawlings, Sarah Rees, Sean Scully, Ting Wang, Hywel T. Evans

**Affiliations:** SAIL Databank, Population Data Science, Swansea University Medical School, Swansea University, Swansea, Wales

**Keywords:** WIMD, Diabetes, Deprivation, Composite measure of deprivation, CMD, Wales, Endogeneity bias, Routine health data, SAIL

## Abstract

**Background:**

The Welsh Index of Multiple Deprivation (WIMD) is an area-based deprivation measure comprising eight domains, produced by the Welsh Government to rank Lower Layer Super Output Areas (LSOAs) in Wales. Researchers use the WIMD to account for deprivation, however, as one domain contains health indicators, there is a risk of endogeneity bias when using the WIMD in research on health outcomes. This study evaluated the effect on study results of removing the health domain from the overall WIMD or using only the income domain as deprivation measures.

**Methods:**

WIMD 2019 scores were linked to 2,760,731 individuals in the SAIL Databank. Original WIMD scores including decile and quintile rankings for each LSOA 2011 were obtained from Welsh Government. The first alternative method removed the health domain from the original WIMD scores. In the second alternative method, WIMD scores were based on only the income domain. Spearman’s correlation and Cohen’s kappa were used to assess the agreement of ranks, deciles, and quintiles between each method. To quantify the change in association between WIMD quintile and diabetes mellitus prevalence for each alternative method, binary logistic regression obtained age-adjusted odds ratios and 95% confidence intervals.

**Results:**

Removing the health domain from the original WIMD scores resulted in 17.28% of LSOAs changing decile (8.64% to a more deprived group and 8.64% to a less deprived group) and 9.00% changing quintile (4.50% more deprived, 4.50% less deprived). The income-domain-only method caused 50.49% of LSOAs to change decile (26.87% more deprived, 23.62% less deprived) as compared with the original WIMD, and 29.65% changed quintile (15.14% more deprived, 14.51% less deprived). There was a significant association between each of the three methods and diabetes prevalence, with odds ratios increasing with more deprived quintiles, but the 95% confidence intervals for each method showed little or no overlap with each other.

**Conclusion:**

To avoid biased estimates, researchers using WIMD in studies on health, education, housing, physical environment, income, employment, community safety, and access to services should consider how these domains are related to their outcomes. We describe a methodology for researchers to quantify any bias in their own studies.

**Supplementary Information:**

The online version contains supplementary material available at 10.1186/s12889-025-22369-0.

## Background

It is well-established that deprivation, including social and economic disadvantages such as poor housing, unemployment, poverty, and a lack of education and opportunities, is associated with poorer health outcomes [1–4]. However, single-item deprivation measures such as household income or employment status may be limited in capturing the multifaceted nature of individual or area-level deprivation [5, 6]. In health research, composite measures of deprivation (CMD) are increasingly being used to control for the effects of individual or area-based deprivation and to assess the effectiveness of interventions aimed at reducing health inequalities [7]. CMDs are constructed by combining a number of different indicators of deprivation, such as income, education, health, and housing, allowing for a more comprehensive assessment of deprivation than single-item measures [6, 8].

Health-related measures of deprivation are increasingly being included in CMDs, but this raises concerns about the possibility of mathematical coupling and the risk of endogeneity bias when using CMDs in studies with a health outcome [7, 9–12]. Endogeneity bias can occur when there is a correlation between an outcome and predictor variable [9]. For example, the Welsh Index of Multiple Deprivation (WIMD) 2019 includes seven health indicators compared to only three in the 2005 release [13, 14]. When Jordan et al. examined the relationship between the English Index of Multiple Deprivation (IMD) 2000 and health inequalities, they hypothesised that the strong correlation between IMD and limiting long-term illness and premature mortality was probably because the IMD included data on the numbers of people claiming benefits for illness and disability [12]. If a CMD that includes indicators of health is used to account for individual or area-based deprivation in a study of the association between an exposure of interest and a health outcome, the potential correlation between the CMD and the outcome may result in over-adjustment, spurious associations, and attenuation or even reversal of the association between the exposure of interest and the outcome.

Recent official guidance on using CMDs in health research cautions against this potential bias [10, 15], yet there is little recent evidence on how different approaches to incorporate CMDs into health research may affect study results. For instance, the Welsh Government guidance on WIMD 2019 encourages researchers interested in the impact of deprivation on health outcomes to recalculate the WIMD to exclude the health domain [15]. However, no published studies to date have quantified the effect on study outcomes of including or excluding a domain of WIMD 2019, which is the gap in knowledge addressed by this study. Gartner et al. examined how excluding the health domain of WIMD 2005 influenced the association between rurality and mortality in Wales [16], but the health domain of the WIMD has changed substantially since the 2005 release [13, 14]. In their study, Gartner et al. found that the choice of deprivation measure influenced the statistical significance of the rurality-mortality relationship but concluded that the observed differences were minor, and attributed them to insufficient statistical power due to the small sample size in Wales [16]. The choice of deprivation measure did not have a significant influence on the rurality-mortality relationship in England, which had a larger sample size [16].

In 2006, Adams et al. assessed the effect of removing the health domain from the 2004 English Index of Multiple Deprivation (IMD) on census measures of health inequalities (limiting long-term illness and less-than-good general health) and found it did not substantially impact socioeconomic inequalities in census measures of health [11]. In a study comparing the overall Scottish Index of Multiple Deprivation to the Scottish Index without the health domain and the income domain in isolation, Bradford and colleagues found that the differences in point estimates for limiting long-term health conditions, self-rated health, and mortality were negligible [10].

Building on previous studies, this study examined the risk of endogeneity bias when using the WIMD in health research using health outcome data from administrative primary care data held in the Secure Anonymised Information Linkage (SAIL) Databank in Wales. This involved a comparison of estimates derived from the overall WIMD 2019, with those obtained from two alternative approaches to incorporating WIMD in health research: (1) WIMD 2019 excluding the health domain, and (2) WIMD 2019 including the income domain only (due to its strong correlation with overall deprivation) [10, 17]. The main aim was to assess the impact of these different approaches on the deprivation ranking of Lower Layer Super Output Areas (LSOA 2011 [18]) in Wales and to quantify the resultant effect on the association between WIMD 2019 quintile and diabetes mellitus prevalence. Diabetes mellitus was chosen as it is defined as one of the GP-recorded chronic conditions in WIMD 2019, where chronic conditions are one of the seven indicators of the health domain (further details given in Table 1). Across the four nations of the United Kingdom (UK), diabetes mellitus had a 7% prevalence in 2019 [19], where around 90% of cases were type 2 which is more common in deprived areas [20]. This study can be used to inform best practice for using CMDs in research and assess the merits of the Welsh Government recommendation [15] to exclude the health domain from WIMD 2019 in research with a health outcome.

### Welsh index of multiple deprivation

The WIMD is a relative measure of deprivation in Wales, focusing on small geographical units (LSOAs) which contain between 1000 and 3000 people, with an average population of around 1600 [14, 21]. The WIMD 2019 comprises of eight distinct domains, each representing a different type of deprivation, including income, employment, health, education, access to services, housing, community safety, and physical environment [14]. It is the Welsh Government’s official measure of relative deprivation for small areas. Based on their relative WIMD score, the LSOAs are ranked from 1 (most deprived) to 1909 (least deprived). Each domain comprises a number of indicators, which are quantifiable measures derived from administrative sources, and less commonly from census data, that assess a particular aspect of deprivation across the 1909 LSOAs in Wales [21, 22]. The indicators are updated periodically and a new WIMD is released every 3 to 5 years. In Table 1, the domains and indicators are based on WIMD 2019 [14, 21].

To determine the WIMD score for each LSOA, the weights assigned by the Welsh Government for each indicator and domain are combined for an overall composite score [21]. The weights were chosen by the Government based on their importance to deprivation in Wales [15, 21]. First, each indicator within the eight domains is assigned a weight. Factor analysis is used to determine weights for the education, health, physical access sub-domain (of the access to services domain), and community safety domains, whereas several methods are used for the others [21]. The weighted sum of domain indicators is ranked, to generate a deprivation ranking for each domain. The domain rankings are then exponentially transformed to create domain scores for each LSOA. The domain scores are weighted according to the respective domain weight, and then aggregated to obtain WIMD index scores for each LSOA. For WIMD 2019, the following domain weights were applied: 22% for income, 22% for employment, 15% for health, 14% for education, 10% for access to services, 7% for housing, 5% for community safety, and 5% for the physical environment [15, 21]. The LSOAs are then ranked by their index scores to assign WIMD ranks, and typically summarised as deciles or quintiles.

**Table 1:** A summary of the eight domains of the Welsh index of multiple deprivation 2019, with information about the indicators in each domain [14, 21, 23]

Domains	Indicators	Calculation of indicators	Data source
Income	An adult, or dependent child of an adult, in receipt of income related benefits	Individuals in each of the four elements are summed (those receiving multiple benefits counted once) and expressed as a percentage of the total residential population for each LSOA, excluding prison populations.	Department for Work and Pensions, His Majesty’s Revenue and Customs, and Home Office.
	An adult, or dependent child of an adult, in receipt of working and child tax credits		
	Supported asylum seeker		
	People on Universal Credit (except those “working with no requirements”)		
Employment	Recipients of job-seekers allowance in working-age population	For each LSOA, the number of working-age residents receiving employment-related benefits is summed (those receiving multiple benefits counted once) and expressed as a percentage of the working-age population, excluding prison populations.	Department for Work and Pensions
	Recipients of employment support allowance in working-age population		
	Recipients of incapacity benefit in working-age population		
	Recipients of Universal Credit and not in employment in working-age population		
Health	People with a GP-recorded diagnosis of a Chronic condition (indirectly age-sex standardised)	The number of chronic conditions, limiting long-term illness, mental health conditions, premature deaths, and cancer incidence are estimated for each LSOA. Individuals with multiple conditions are counted once. Obesity in 4-5-year-olds is calculated using age and gender-specific BMI centiles, with those at or above the 95th centile classified as obese.	Digital Health and Care Wales, Office for National Statistics (ONS), Velindre NHS Trust, and Public Health Wales
	People with a GP-recorded diagnosis of a Mental health condition (indirectly age-sex standardised)		
	Cancer Incidence (indirectly age-sex standardised)		
	Limiting Long-Term Illness (indirectly age-sex standardised)		
	Premature Death Rate (death of those under the age of 75)		
	Children aged 4–5 who are Obese		
	Low Birth Weight, single births (live births less than 2.5 kg)		
Education	Foundation phase average point score	The Foundation Phase average point score, KS2 average point score, KS4 average point score for core subjects, repeat absenteeism, and KS4 leavers entering higher education are three-year averages of pupils’ scores, highest scores in core subjects, percentages missing over 15% of sessions, and percentages entering higher education within three years of leaving Year 11, respectively.	NPD, Pupil Level Annual School Census (PLASC), NDC, Higher Education Statistics Authority (HESA) Record, Lifelong Learning Wales Record (LLWR), WED, Census, and ONS
	Key stage 2 (KS2) average point score		
	Key stage 4 (KS4) average point score for core subjects		
	Repeat absenteeism		
	Proportion of key stage 4 leavers entering higher education		
	Number of adults aged 25–64 with no qualifications		
Access to Services	Average public and private travel time to Pharmacy	Physical access to services is estimated as the weighted average travel times to eight services by public transport and nine by private transport. Public times are calculated using the propeR tool, and private times use the pgRouting library in PostGIS with average vehicular speeds from Basemap. Digital access is the percentage of homes and small businesses that cannot receive 30 Mb/s fixed line broadband.	Welsh Government and Office of Communications (Ofcom)
	Average public and private travel time to Food shop		
	Average public and private travel time to General Practitioner (GP)		
	Average public and private travel time to Post office		
	Average public and private travel time to Primary school		
	Average public and private travel time to public library		
	Average public and private travel time to Sports Facility		
	Average public and private travel time to Secondary school		
	Average private travel time to Petrol station		
	Digital Access (percent unavailability of broadband at 30 Mb/s)		
Housing	Percentage of people living in overcrowded households (bedrooms measure)	Overcrowding is assessed by calculating a household’s required bedrooms based on the ages and relationships of household members and subtracting it from the actual number of bedrooms to determine the occupancy rating. Poor quality housing is determined by a model that combines survey and administrative data to predict which dwellings are likely to be in disrepair or contains serious hazards.	Census, ONS, and Building Research Establishment
	Likelihood of poor-quality housing		
Community Safety	Police recorded criminal damage	The community safety domain is calculated as the number of burglaries, fire, theft, violence, anti-social behaviour, and criminal damage offences in a given area averaged over two years, with the denominator the number of people or properties in that area.	Welsh Police Forces, Incident Recording System (IRS), and Welsh Government
	Police recorded violent crime		
	Police recorded anti-social behaviour		
	Police recorded burglary		
	Police recorded theft		
	Fire incidents		
Physical Environment	Population weighted average concentration values of Nitrogen dioxide (NO_2_)	The three air quality indicators are measured using data on the average pollutant concentration for every square km in the UK to assign NO2, PM10, and PM2.5 levels to dwellings in Wales, which is then averaged for LSOAs. Flood risk indicator assesses the proportion of households at risk of flooding from rivers, sea, and surface water as low, medium, or high based on predicted frequency, not damage level. Proximity to green space is determined by combining Open Greenspace data and natural greenspace typologies to estimate dwellings within 300 m of green space, which are then aggregated at the LSOA. The ambient Green Space indicator is calculated as the Mean Normalised Difference Vegetation Index (NDVI) within a 300-metre Euclidean buffer around each residential dwelling.	Department for Environment, Food & Rural Affairs (DEFRA), ONS, Flood Risk Assessment Wales data, Natural Resources Wales, OS MasterMap Topography Layer, AddressBasePlus, and Administrative Data Research (ADR-Wales)
	Population weighted average concentration values of Particulates < 10 μm (PM_10_)		
	Population weighted average concentration values of Particulates < 2.5 μm (PM_2.5_)		
	Flood risk		
	Proximity to accessible, natural green space		
	Ambient green space score		

### Methods

We obtained data for the WIMD 2019 domain scores for each LSOA 2011 [18] from the Welsh government website [24, 25]. Weighted domain scores were calculated using the 2019 WIMD domain weights, then three different methods were applied:

1. Method one: Following the Welsh Government’s original method, the weighted domain scores were summed to produce the WIMD score for each LSOA. The overall scores were ranked, and then grouped into decile (1– most deprived, 10– least deprived) and quintile (1– most deprived, 5– least deprived) categories, as is available from Welsh Government [24, 25].
2. Method two: In the second method, to construct the ‘WIMD-minus-health’ scores, we excluded the health domain and recalculated the WIMD scores, ranks, deciles, and quintiles for each LSOA using the summed weighted scores of the remaining seven domains.
3. Method three: In the third method, we constructed rankings, deciles, and quintile groupings for the ‘income-domain-only’ method using only the income domain score of the eight WIMD 2019 domains.

The Spearman’s rank correlation coefficient was used to assess the correlation between the deprivation rankings, deciles, and quintile groupings for the original WIMD with each of the two alternative deprivation calculation methods (WIMD-minus-health, and income-domain-only). We used Cohen’s kappa statistic with equal weighting [26] to assess the agreement between the deprivation rankings, deciles, and quintile groups produced by the three methods. In addition, we quantified the change in LSOA decile and quintile groupings as a result of the two alternative WIMD methods, and plotted these changes using histograms in R.

To estimate the effect of excluding WIMD domain scores on diabetes prevalence, the data for the original WIMD 2019 and the alternative methods were linked at the LSOA level with individual-level routine health data for all-time diabetes mellitus diagnoses in the SAIL Databank, using the LSOA of residence on 01 January 2019 (the census date of this study). The SAIL Databank is a secure repository of linked anonymised health and administrative data in Wales, which is accessible via a trusted research environment [27–29]. Within SAIL, anonymised individuals in Wales with a diabetes mellitus diagnosis were flagged using a published code list [30] and the Welsh Longitudinal General Practice (WLGP) dataset, which contains primary care records for 86% of individuals in Wales. We used binary logistic regression adjusted for age at the census date to assess the relationship between the lifetime prevalence of diabetes mellitus and ranked quintile groupings for original-WIMD, WIMD-minus-health, and income-domain-only. Odds ratios with 95% confidence intervals were calculated, and the analysis was conducted separately for males and females in a sex-stratified approach.

## Results

**Fig. 1:**
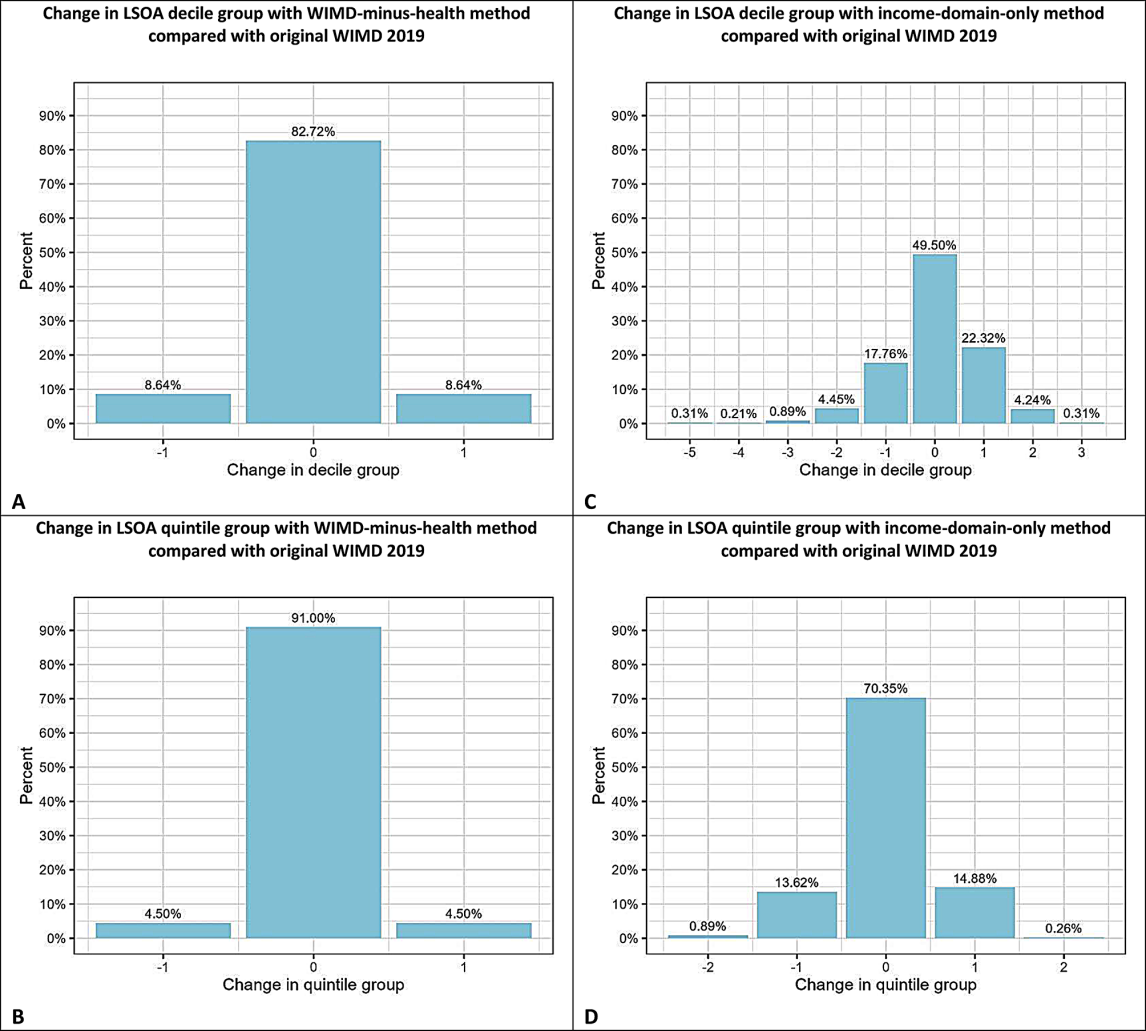
Changes in WIMD 2019 decile and quintile groups for Lower Layer Super Output Areas (LSOA 2011) following modification of the original WIMD for the WIMD-minus health method (**A** and **B**) and income-domain-only method (**C** and **D**). A negative difference indicates a move to a less deprived group than the original group, while a positive difference represents a move to a more deprived group

The analytical sample consisted of 2,760,731 individuals alive in the Welsh Demographic Service Dataset (WDSD) in the SAIL Databank on 01 January 2019 (the census date). 49.75% were male and 50.25% were female. The median age of the population was 40 years (male: 39 years, female: 41 years) and the mean age was 40.0 years (standard deviation (SD): 23.6 years) with a male mean age of 39.2 years (SD: 23.3 years) and female mean age of 40.8 years (SD: 23.9 years). The percentage of the study population in each WIMD quintile was between 19% and 21%, showing good representativeness of the Welsh population (values in Supplementary Table 1). Figure 1 shows the changes in ranked LSOA 2011 decile and quintile groups following the two alternative methods.

For method two, WIMD-minus-health, 82.7% of LSOAs remained in the same decile group, but 17.3% of LSOAs changed decile group when the health domain was excluded. Of these, 8.6% moved one decile to a more deprived rank, and 8.6% moved one decile to a less deprived rank. When considering quintile WIMD groups, 91.0% of LSOAs remained within their initial quintile, but 9.0% changed quintile group, with 4.5% of these moving one quintile to a more deprived quintile, and the other 4.5% moving one quintile to a less deprived quintile.

In method three, income-domain-only, only around half (49.5%) of the LSOAs kept their original decile grouping. Among the 50.5% of LSOAs that changed decile rank, 26.9% moved to more deprived decile groups, while 23.6% moved to less deprived decile groups, with six (0.3%) LSOAs moving as much as five decile groups. When considering quintile groupings, the majority (70.3%) of LSOAs remained in the same quintile group. However, 29.7% of LSOAs changed quintile, with 13.6% and 0.9% moving one and two quintiles to a less deprived rank and 14.9% and 0.3% moving one and two quintiles to a more deprived rank, respectively.

To quantify the agreement between the original-WIMD 2019 and the two alternative methods we calculated correlation and Kappa agreement scores, along with the proportion of variance explained for each (Table 2). There was close agreement between the alternative methods and the original-WIMD 2019, but with higher correlation and kappa values for WIMD-minus-health than income-domain-only. WIMD-minus-health explained 99.3% of the variance in WIMD2019, and the correlation and kappa coefficients, comparing quintiles of WIMD-minus-health with those of original-WIMD 2019, showed very close agreement (rank [correlation = 99.6%, Kappa_w_ = 99.6%]; decile [correlation = 99.0%, Kappa_w_ = 99.0%]; and quintile [correlation = 97.7%, Kappa_w_ = 97.7%]). The Income-domain-only method explained less, only 93.9%, of the variance in original-WIMD, and agreement between the income-domain-only and original-WIMD was close, but less than the WIMD-minus-health method (rank [correlation = 95.0%, Kappa_w_ = 95.0%]; decile [correlation = 94.1%, Kappa_w_ = 94.1%]; and quintile [correlation = 91.7%, Kappa_w_ = 91.7%]).

**Table 2:** Correlation and agreement for the LSOA ranks, decile, and quintile groups between the original-WIMD 2019 and the WIMD-minus-health method, and between the original-WIMD 2019 and the income-domain-only method (95% confidence intervals are shown in brackets)

	WIMD-minus-health vs. original-WIMD 2019	Income-domain-only vs. original-WIMD 2019
Proportion of variance explained	0.9927	0.9391
	**LSOA Rank**	
Spearman’s correlation	0.9960 (0.9954–0.9964)	0.9500 (0.9423–0.9560)
Cohen’s Kappa_w_	0.9961 (0.9956–0.9965)	0.9500 (0.9439–0.9561)
	**LSOA Decile**	
Spearman’s correlation	0.9895 (0.9882–0.9907)	0.9412 (0.9332–0.9474)
Cohen’s Kappa_w_	0.9895 (0.9883–0.9907)	0.9412 (0.9348–0.9476)
	**LSOA Quintile**	
Spearman’s correlation	0.9775 (0.9738–0.9808)	0.9172 (0.9092–0.9245)
Cohen’s Kappa_w_	0.9775 (0.9740–0.9809)	0.9172 (0.9093–0.9251)

**Fig. 2:**
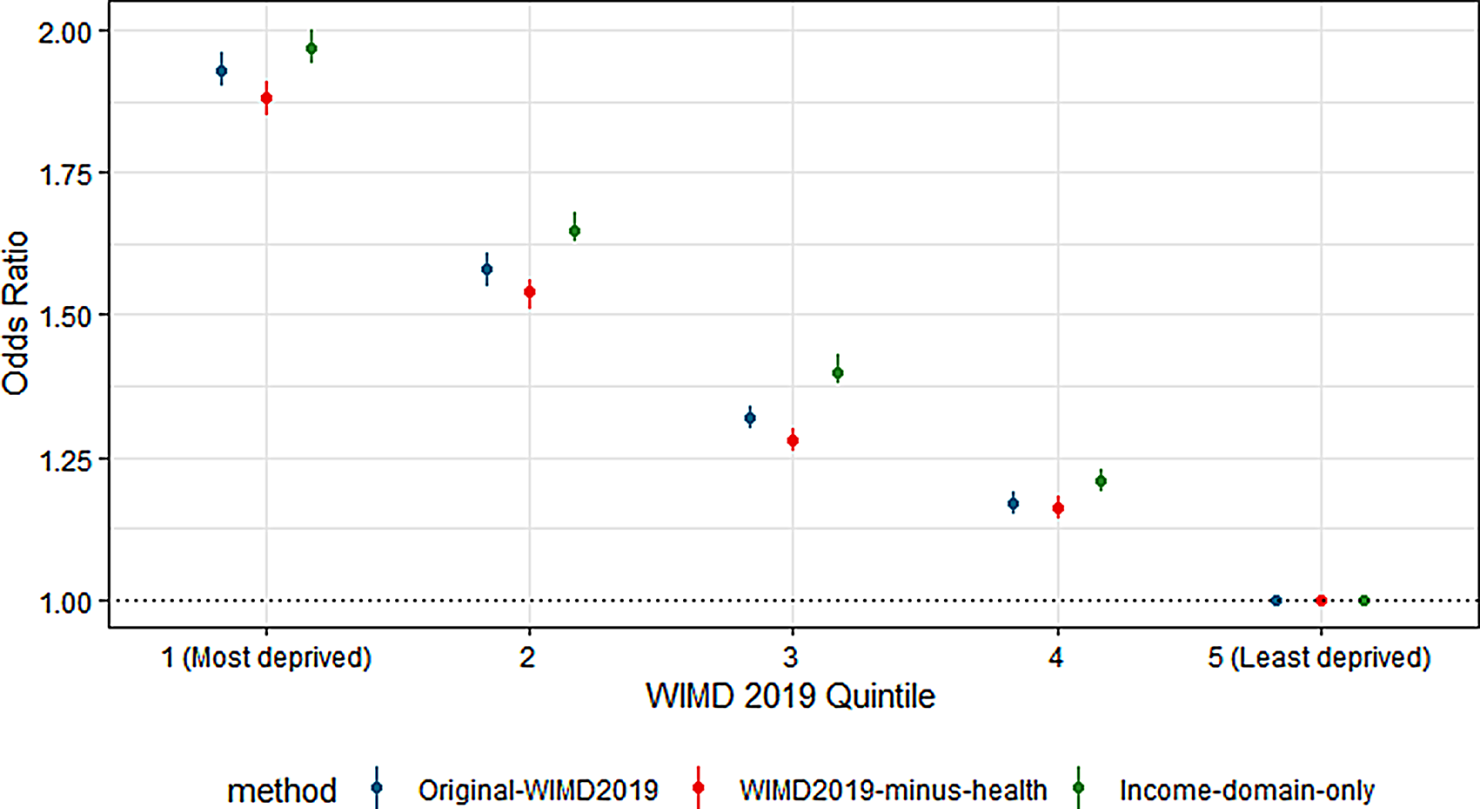
Age-adjusted odds ratios quantifying the association between WIMD 2019 quintile and the prevalence of diabetes mellitus in primary care, for the three WIMD methods: original-WIMD2019, WIMD2019-minus-health, and income-domain-only. 95% confidence intervals are shown, and the reference group is WIMD quintile 5 (least deprived)

In this study sample, 6.5% (178,451 people) of the participants had a diabetes mellitus diagnosis, in agreement with the 6.5% reported for Wales [31] and the 7% across the UK, which was broadly the same across the four nations [19]. Figure 2 shows age-adjusted odds ratios and 95% confidence intervals for the prevalence of diabetes mellitus in primary care, stratified by WIMD 2019 quintiles based on: [1] original-WIMD2019 [2], WIMD2019-minus-health, and [3] income-domain-only. The results of the original WIMD 2019 analysis, which included the health domain, show a significant association between deprivation quintile and diabetes prevalence, with the odds ratios for diabetes increasing with deprivation. The second method, WIMD-minus-health, also showed a strong positive association between diabetes prevalence and deprivation quintile but with lower odds ratios compared with the original-WIMD. In the third method, estimates using income-domain-only also show a significant positive association between income-based deprivation and diabetes prevalence. In this case, the odds ratios for each quintile were higher than those from both the original-WIMD and the WIMD-minus-health. The sex-stratified results in Table 3 show similar associations and patterns between the deprivation quintiles of the three WIMD 2019 methods and diabetes prevalence.

**Table 3:** The impact of the different methods of incorporating WIMD on a health outcome (diabetes mellitus). The total sample contained 2,760,731 individuals. Odds ratios quantifying the association between WIMD 2019 quintile and the risk of diabetes mellitus from binary logistic regression models for each of the three methods are shown, also stratified by sex. Results were adjusted for age on 01 January 2019, and the reference group is WIMD quintile 5 (least deprived)

Diabetes mellitus						
	Total sample		Male		Female	
	Number with diabetes (%)*	OR (95% CI)	Number with diabetes (%)*	OR (95% CI)	Number with diabetes (%)*	OR (95% CI)
**WIMD 2019 quintile**						
1 (Most deprived)	41,515 (7%)	1.93 (1.90–1.96)	22,258 (8%)	1.76 (1.72–1.80)	19,257 (7%)	2.16 (2.11–2.21)
2	39,394 (7%)	1.58 (1.55–1.61)	21,805 (8%)	1.51 (1.47–1.54)	17,589 (6%)	1.69 (1.65–1.73)
3	34,908 (6%)	1.32 (1.30–1.34)	19,786 (7%)	1.28 (1.25–1.30)	15,122 (6%)	1.38 (1.34–1.41)
4	32,159 (6%)	1.17 (1.15–1.19)	18,366 (7%)	1.15 (1.12–1.18)	13,793 (5%)	1.21 (1.18–1.24)
5 (Least deprived)	30,475 (6%)	1.00	17,753 (6%)	1.00	12,722 (5%)	1.00
**WIMD-minus-health quintile**						
1 (Most deprived)	41,391 (7%)	1.88 (1.85–1.91)	22,197 (8%)	1.72 (1.69–1.76)	19,194 (7%)	2.10 (2.05–2.15)
2	38,911 (7%)	1.54 (1.51–1.56)	21,628 (8%)	1.47 (1.44–1.50)	17,283 (6%)	1.63 (1.59–1.67)
3	34,149 (6%)	1.28 (1.26–1.30)	19,310 (7%)	1.24 (1.21–1.27)	14,839 (6%)	1.34 (1.31–1.37)
4	33,011 (6%)	1.16 (1.14–1.18)	18,857 (7%)	1.14 (1.12–1.17)	14,154 (5%)	1.19 (1.16–1.22)
5 (Least deprived)	30,989 (6%)	1.00	17,976 (7%)	1.00	13,013 (5%)	1.00
**Income-domain-only quintile**						
1 (Most deprived)	39,053 (7%)	1.97 (1.94–2.00)	20,904 (8%)	1.81 (1.77–1.85)	18,149 (7%)	2.21 (2.16–2.26)
2	39,151 (7%)	1.65 (1.63–1.68)	21,583 (8%)	1.58 (1.54–1.61)	17,568 (6%)	1.78 (1.74–1.83)
3	36,718 (7%)	1.40 (1.38–1.43)	20,727 (8%)	1.37 (1.34–1.40)	15,991 (6%)	1.47 (1.43–1.50)
4	34,477 (6%)	1.21 (1.19–1.23)	19,689 (7%)	1.17 (1.15–1.20)	14,788 (5%)	1.25 (1.22–1.29)
5 (Least deprived)	29,052 (5%)	1.00	17,065 (6%)	1.00	11,987 (4%)	1.00

* n (%) = the number and row percentage of people with diabetes mellitus in each WIMD 2019 quintile

## Discussion

Our study examined the potential risk of endogeneity bias associated with using the Welsh Index of Multiple Deprivation (WIMD) in health research and demonstrates changes in LSOA rankings with the exclusion of different domains from the index. We suggested two alternative methods to reduce the risk of potential endogeneity bias associated with using the original WIMD 2019 in health research: the WIMD-minus-health (method two) and income-domain-only (method three). Both alternative methods caused changes in the LSOA deprivation deciles and quintile groupings, despite high overall agreement with the original WIMD 2019. Method two demonstrated closer agreement with method one than method three, as method two retained seven of the original eight domains, whereas method three included only one of the eight domains. The relatively high level of agreement between the original WIMD and the two alternative methods supports that these proposed alternatives could be used instead of the original WIMD 2019, where research with the health outcome suggests endogeneity bias.

A strong positive association was found between the WIMD 2019 deprivation quintiles and diabetes prevalence, in line with previous studies [32]. However, the confidence intervals of the odds ratios using the WIMD-minus-health and income-domain-only methods had no overlap, with only a small overlap with the original WIMD 2019. The difference between these estimates demonstrates that results are sensitive to the choice of deprivation method and exclusion of WIMD domain(s). For example, when the health domain was excluded, WIMD-minus-health maintained a strong positive association with diabetes, but the estimates attenuated as compared to the original WIMD, aligning with concerns of overestimation associated with the inclusion of health-related indicators in composite deprivation measures in health research. We advise that in most cases, researchers should follow method two and remove domains that are related to their study outcomes. However, the income-domain-only method produced higher odds ratios than both the original WIMD and WIMD-minus-health, suggesting a stronger association between diabetes and area-based income deprivation. This finding also demonstrates the potential use of the income domain as a more succinct method of representing deprivation, particularly when the focus is on capturing socioeconomic deprivation. Our findings provide empirical support for the Welsh Government’s cautionary guidance on using WIMD in health research [15] and highlight the need for researchers to carefully consider the components of composite deprivation measures based on their study context.

In line with our results, Bradford et al. observed a high level of agreement when comparing the overall 2012 and 2020 Scottish Index of Multiple Deprivation (SIMD) with the health-excluded and income-domain-only methods [10]. Adams and White also found strong agreement between the English IMD 2004 and IMD 2004-minus-health [11]. In Bradford et al.‘s study, excluding the health domain of SIMD resulted in 17.6% and 13.5% of areas shifting to lower or higher deprivation quintiles respectively, which aligns with our findings [10]. Our results are also consistent with the concordance reported by Gartner et al., in their comparison of alternative methods with WIMD 2005 [16]. Furthermore, a previous comparison revealed that the income domain of the Indices of Multiple Deprivation for England, Northern Ireland, Scotland, and Wales explained at least 94% of the variation in each country’s overall deprivation index [7], consistent with our findings.

However, in our study, excluding the health domain and using only the income domain had a greater effect on results than in previous studies, especially for the income-only method. In Gartner et al.‘s [16] study of WIMD 2005, alternative methods influenced the statistical significance of the results, but the changes to the estimates were small as compared to the differences seen in our study. For health and mortality outcomes, Bradford et al., found only slight differences in point estimates between the overall SIMD, SIMD-minus-health, and income-only deprivation methods [10]. Adams and White [11] also concluded that removing the health domain of the English IMD had minimal practical effect on measured socioeconomic inequalities in census-based health measures.

Our study may have found a larger effect than Gartner et al.‘s earlier study in Wales [16] due to differences in the health domain indicators between WIMD 2005 and WIMD 2019. The WIMD 2005 [33] health domain did not include indicators for chronic conditions, mental health conditions, child obesity, and low birth weight. Furthermore, it has been shown that deprivation correlates more strongly with long-term limiting illnesses (such as diabetes) than with mortality [12], which further explains why our study demonstrated a larger effect compared to Gartner et al.’s study, in which the outcome of interest was mortality. Additionally, in WIMD 2019, the health domain has a weight of 15%, whereas in the Scottish and English deprivation measures, the domain has a weight of 14% and 13.5%, respectively. The lower weight in the Scottish and English deprivation measures, relative to WIMD 2019, might have contributed to the smaller differences observed in the impact of excluding the health domain in SIMD and English IMD.

Other variations in findings can be attributed to differences in data sources and population characteristics across the studies. The outcomes analysed in the previous studies were self-reported limiting long-term illness, self-rated general health, and all-cause mortality, all obtained from census data, which would result in a difference, as our outcome was GP-diagnosed diabetes from primary care sources. Therefore, bias due to misclassification would be minimal in our study outcome compared to the previous studies. Furthermore, the impact of deprivation on health outcomes differs in Wales compared to other UK countries [7]. A study of WIMD 2005 and IMD 2004 by Gartner et al. showed that the impact of the deprivation methods and their alternative forms on mortality differed between nations [16]. Thus, these existing disparities also contribute to the larger effect seen in our study, when compared to those from Scotland and England.

In contrast to earlier studies that relied on self-reported health outcomes, our study’s results were based on a GP-diagnosed health condition derived from a dataset covering 86% of the population in Wales, thereby improving the generalisability of our results. Diabetes mellitus is a monitored condition under the Quality Outcomes Framework (QOF) in Wales, which forms part of the General Medical Services contract, therefore diagnoses should be well-recorded by GPs in the WLGP dataset. Our study also had a relatively large sample size, and we were able to link to up-to-date administrative data, which was an improvement compared with previous research. However, the focus on a single health outcome is the key limitation of this study, as other health conditions will show different patterns, and it may not capture the complexity of more sophisticated health studies. For this reason, researchers should undertake their own preliminary exploration for their studies. Additionally, our findings are context-specific to Wales, and WIMD is area-based rather than individual-level, so caution should be exercised in generalising to other populations at different times.

Future studies should examine the potential bias associated with including or excluding other domains of the WIMD. For example, the education domain for some LSOAs may be more sensitive due to a smaller number of individuals contributing to the education domain score, as compared to the number of people with chronic health conditions. Evaluating different outcomes and data sources to provide a more comprehensive understanding of how WIMD impacts various study outcomes is worthwhile. To enable this, the methodology and scripts used in this work will be made available from the SAIL Databank and can be used as a basis for future methodological enhancements.

## Conclusion

We highlighted the effect of ignoring potential endogeneity bias in research when using measures of multiple deprivation and offered methodology to mitigate this bias. The strength of this bias and the effect of mitigation will differ depending on the specific cohort or outcomes of interest, but an example has been evaluated using a reproducible methodology. The number of LSOAs affected by removing a domain from the WIMD 2019 was reported, along with demonstrating that the strength of association between WIMD and a health outcome can be significantly affected by the choice of deprivation measure. We utilised a highly representative sample of the Welsh population from the SAIL Databank, whereas other studies will typically have more specific cohort exclusion criteria and may be less representative. Consequently, the results of this study may underestimate or overestimate this source of endogeneity bias, as it is dependent upon the number of individuals in LSOAs that change WIMD rank when a domain is removed, and the strength of the association between WIMD rank and the outcome of interest. To uphold scientific rigour, future studies utilising the WIMD in health, education, housing, physical environment, income, employment, community safety, and access to services related research should consider these implications. We have provided the methodology and tools for researchers to assess and quantify the effect of this commonly overlooked source of bias for their own studies.

## Electronic supplementary material

Below is the link to the electronic supplementary material.

Supplementary Material 1

## Data Availability

Data on the Welsh Index of Multiple Deprivation (WIMD) are publicly available at the Welsh government’s official website (https://www.gov.wales/welsh-index-multiple-deprivation-full-index-update-ranks-2019). Data on diabetes mellitus diagnoses from the Welsh Longitudinal General Practice dataset (WLGP) are available via the SAIL Databank, and requests to access data are reviewed by the SAIL independent Information Governance Review Panel. The data analysed in this study (SAIL project 1480) are available from the SAIL Databank, following additional approval by the corresponding author on reasonable request.
