# Supplementary material for: Using the Welsh Index of Multiple Deprivation in research: estimating the effect of excluding domains on a routine health data study
Source: BMC Public Health. 2025 Mar 28;25:1178. doi: 10.1186/s12889-025-22369-0 (PMC11951554; doi:10.1186/s12889-025-22369-0)
Supplement: Supplementary file 1 — Supplementary Material 1 [file 12889_2025_22369_MOESM1_ESM.docx]

**Supplementary Table 1: Count and column percentage of people in each original WIMD 2019 quintile, for the total study population and those with a diabetes mellitus diagnosis.**

| **WIMD 2019 Quintile** | **Total study population n (%)** | **Diabetes population**  **n (% of total diabetes population)** |
| --- | --- | --- |
| 1 (Most deprived) | 583,168 (21%) | 41,515 (23%) |
| 2 | 562,032 (20%) | 39,394 (22%) |
| 3 | 542,476 (20%) | 34,908 (20%) |
| 4 | 519,146 (19%) | 32,159 (18%) |
| 5 (Least deprived) | 553,909 (20%) | 30,475 (17%) |
